# Adherence to oral thromboprophylaxis in atrial fibrillation: an overview for clinicians

**DOI:** 10.1093/europace/euaf250

**Published:** 2025-10-13

**Authors:** Tatjana Potpara, Bogdan G Markovic, Marek Grygier, Simonetta Genovesi, Apostolos Tzikas, Serge Boveda, Jens Erik Nielsen-Kudsk, Giuseppe Boriani, Gregory Y H Lip, A John Camm

**Affiliations:** Medical Faculty, University of Belgrade, Dr Subotica starijeg 8, Belgrade 11000, Serbia; Cardiology Clinic, University Clinical Centre of Serbia, Dr Subotica starijeg 13, Belgrade 11000, Serbia; Medical Faculty, University of Belgrade, Dr Subotica starijeg 8, Belgrade 11000, Serbia; 1st Department of Cardiology, Poznan University School of Medical Sciences, Poznan, Poland; School of Medicine and Surgery, University of Milano-Bicocca, Nephrology Clinic, Monza, Italy; Istituto Auxologico Italiano, IRCCS, Milan, Italy; Ippokrateio Hospital of Thessaloniki, Aristotle University of Thessaloniki, Thessaloniki, Greece; Structural and Congenital Heart Disease, European Interbalkan Medical Centre, Thessaloniki, Greece; Cardiology, Heart Rhythm Management Department, Clinique Pasteur, Toulouse, France; Cardiologie Clinique Pasteur, Brussels University VUB, Brussels, Belgium; Department of Cardiology, Aarhus University Hospital, Aarhus, Denmark; Cardiology Division, Department of Biomedical, Metabolic and Neural Sciences, University of Modena and Reggio Emilia, Policlinico di Modena, Modena, Italy; Liverpool Centre for Cardiovascular Science at University of Liverpool, Liverpool John Moores University and Liverpool Heart & Chest Hospital, Liverpool, UK; Danish Center for Health Services Research, Department of Clinical Medicine, Aalborg University, Aalborg, Denmark; Genetic and Cardiovascular Sciences Institute, Cardiology Academic Group, St. George’s University of London, Cranmer Terrace, London SW19 0RE, UK

**Keywords:** Atrial fibrillation, Oral anticoagulant therapy, Adherence to medication, Stroke, Bleeding

## Abstract

In most patients with atrial fibrillation (AF), effective stroke prevention necessitates long-term (often lifelong) oral anticoagulant therapy (OAC). However, the effectiveness of OAC therapy in a clinical setting (i.e. outside the controlled environment of randomized clinical trials) is strongly influenced by patients’ adherence and persistence with prescribed therapy. However, suboptimal adherence to OAC remains a substantial problem in routine practice—available evidence suggests that patients do not take their OAC one out of every four days, and approximately one in three to four patients is poorly adherent to OAC. In addition, around 15% of high-risk OAC-eligible patients with AF refuse to take OAC for a variety of patient-specific reasons. Poor adherence to OAC therapy is associated with adverse clinical outcomes [such as stroke or systemic embolism, hospitalization, mortality, bleeding (particularly with vitamin K antagonist therapy)] and increased economic costs. In this overview, we summarize important aspects of the adherence to medication concept, including the definition and measurement of adherence, the determinants and prevalence of OAC non-adherence, the clinical importance of achieving and maintaining good adherence, strategies to improve adherence to OAC, and alternative treatment options for effective thromboprophylaxis in patients with AF who are non-adherent to OAC therapy.

## Graphical Abstract

**Graphical Abstract:**
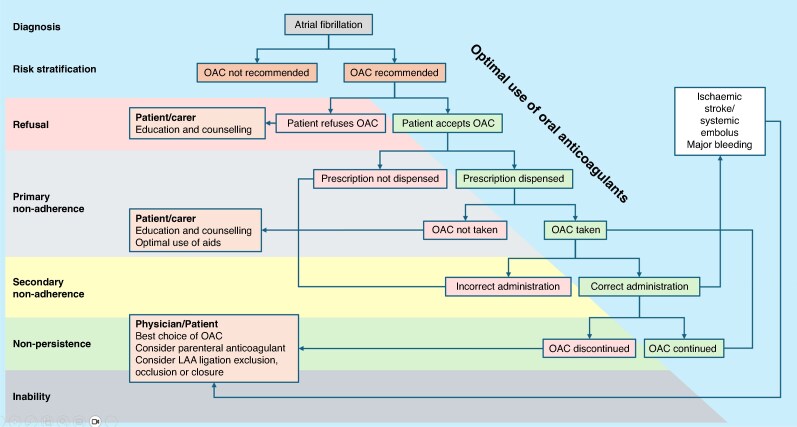

## Introduction

Oral anticoagulant therapy (OAC) using vitamin K antagonists (VKA) or, preferably, direct oral anticoagulants (DOACs) is recommended in guidelines as a cornerstone of optimal thromboprophylaxis in patients with atrial fibrillation (AF).^1,2^ In most patients with AF, effective stroke prevention necessitates a long-term (often lifelong) OAC therapy. However, the effectiveness of outpatient OAC therapy in a clinical setting [that is, outside the controlled environment of randomized clinical trials (RCTs)] is strongly impacted by patients’ adherence and persistence to prescribed therapy, in addition to physician’s adherence and commitment to guideline recommendations.^3^ Notably, the effectiveness of DOAC is potentially more sensitive to suboptimal adherence compared with VKAs, given their short half-lives,^4^ whereas adherence to VKAs also requires periodic International Normalized Ratio (INR) measurement.^1,2^

Adherence to OAC is teamwork dependent that requires a multidisciplinary approach with constant active engagement of the responsible physician(s) and healthcare professional(s), the patient, and the patient’s family members or carers. Multiple determinants of poor adherence have been identified, related to the patient, the medication itself, overall medical condition, or the patient/physician relationship and the healthcare system, and the financial circumstances of the patient,^5^ Various strategies for maintaining good adherence to OAC therapy have been explored,^6^ but suboptimal adherence to OAC is still a substantial problem in routine practice.^3,5^

Poor adherence to OAC therapy is associated with adverse clinical outcomes [such as stroke or systemic embolism, hospitalization, mortality, bleeding (particularly with VKA therapy)] and increased economic costs.^3^

In this overview, we summarize important aspects of the adherence to medication concept, including the definition and measurement of adherence, the determinants and prevalence of OAC non-adherence, the clinical importance of achieving and maintaining good adherence, strategies to improve adherence to OAC, and alternative treatment options for effective thromboprophylaxis in patients with AF non-adherent to OAC therapy.

## Definition of adherence to medication

*Adherence* to medication refers to the extent of following prescribed instructions on dose, intake intervals, and treatment duration when taking a medication.^7,8^

While adherence implies an active patient engagement, including agreement to the treatment plan with the responsible physician. The term *compliance* with medication is less preferred since it possibly connotes a more passive patient role.^8,9^ Finally, the term *persistence* to medication complements adherence, since it concerns the continuity of adherence to medicines within a particular period.^8^

**Figure 1:**
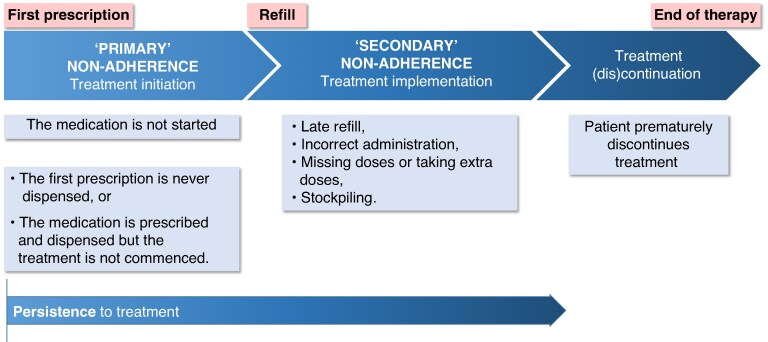
The concept of adherence to medication.^10,11^

Medication adherence research mostly focuses on so-called ‘secondary’ adherence (i.e. the treatment implementation) and persistence, whereas ‘primary’ adherence (i.e. the prescription for medication or its appropriate alternative never filled/dispensed and/or the treatment never commenced) is less well studied^10^ (*Figure 1*).

### Patient refusal

Some patients are not willing to take the prescribed medication even when provided with a detailed explanation of the net clinical benefit of the proposed therapy. Patient refusal may underlie either primary or secondary non-adherence/non-persistence to medication.

Patient refusal to medication may arise from a variety of reasons (see the sections below) and, sometimes, patients may revise their initial non-acceptance of proposed treatment after reconsideration of risks and benefits. However, the informed patient’s refusal usually represents an ultimate form of non-adherence to medication, necessitating the consideration of viable alternatives where possible.

## Assessment of adherence to medication

There are multiple methods for measuring medication adherence.^7–9,11–13^ The choice of a specific method should consider the research question(s) of interest, available data source(s) and feasible study design(s). When interpreting the findings, both the strengths and disadvantages of the specific approach, with the possibility of either under- or overestimation of adherence, should be considered (*Table 1*).

**Table 1:** Examples of various approaches to the assessment of medication adherence

Method	Measure	Description	Strengths	Limitations
Retrospective	Medication possession ratio (MPR)^8,14^	The number of days the patient has access to the medication divided by the number of days in the period The MPR values typically fall between 0 and 1. When patients refill their prescription before completing their previous supply, MPR is >1	Retrospective dataset availability Sample size (large samples improve study power and external validity) ‘Real-world’ setting (facilitates generalizability) Usually less costly	Potentially inaccurate or missing data (e.g. free samples and medication paid by the patient is not captured) Selection bias (uninsured patients or those with a different insurance plan may be different from patients included in the study) Uncertain correlation(s) between measures of medication possession and adherence to medication
	Proportion of days covered (PDC)^8,15^	Number of days with medication supply available (i.e. days ‘covered’) divided by the total number of days in the period If there is an overlap in supply, the numerator should be adjusted to reflect the offset of days covered		
	Group-based trajectory modelling (GBTM)^16–18^	Categorizing patients with comparable medication filling patterns into clusters Incorporating GBTM into regression models may identify cluster-specific patient characteristics and outcomes or predict patterns of non-adherence		
Prospective	Direct observation^19^	Watching patients while they use their medication and/or observing the anticipated effects of the medication	Accurate confirmation that a medication has been taken/administered	Time-consuming Needs excessive labour (especially with a long-term study follow-up) Costly Subjected to manipulation by patients (does not account for medications patient does not bring to the visit or has wasted)
	Pill count^8,19^	Days supplied are combined with periodic counts of remaining units of medication. Allows observing the number of medications in a blister pack, the level of liquid in a bottle, or the number of doses remaining in an inhaler		
	Therapeutic drug monitoring^8^	Biological assays to measure the concentration of drugs, metabolites, or biomarkers in the patient’s serum, plasma, or other fluids Timing and frequency of measurement, as well as the choice of specific assays, depend on the pharmacokinetic properties of the medication^a^	Accurate and objective measurable confirmation of medication use	Costly Invasive Dependent on patient-specific factors Requires proper assay(s) The risk of missing data
	Devices^8,20^	Electronic pill boxes, pill bottle/inhaler/nebulizer monitors, and swallowable devices that provide information on when and how the device is used	Real-time data collection without direct monitoring Observation of the timing of medication use	Costly Technology-specific issues Do not guarantee that a medication has been ingested
Patient or clinician reported	Diaries^21,22^	Real-time capturing of events of interest, such as timing of medication intake, missed dose(s), challenges related to medication administration, side effects, etc.	Real-time data collection Higher accuracy and lower recall bias Lower costs	Greater burden to patients Reporting and performance bias Misreporting Missing data
	Unstructured or semi-structured interviews^8,21^	Personalized assessment of reasons underlying (non)medication adherence. No need for access to insurance claims data or medical records	Allow using personalized questions Provide ‘subjective’ data for qualitative (or semi-qualitative) studies	Usually not validated Recall and/or interviewer bias Social desirability bias Possibly limited generalizability
	Structured survey instruments^23–32^	The use of a validated instrument that enables the collection of standardized quantitative data, which can be used for comparisons between subjects or cohorts	Validated tools Usually low cost	Specific tools may require fees or permission for use Risk of survey fatigue Response/recall/reporting/social desirability bias Unanswered questions/missing data

^a^Of note, the use of serum anti-Xa levels measurement currently is not recommended for routine assessment of adherence to DOAC in clinical practice^1,2,4^ for several reasons. Most importantly, since DOACs, unlike vitamin K antagonists, have short half-lives, plasma levels fluctuate significantly over a dosing interval, and a single anti-Xa level at any given time reflects current drug activity, not past adherence. Whereas a single measurement of undetectable or very low DOAC levels do not prove long-term non-adherence to DOAC, serial measurement of DOAC trough levels could provide adherence estimates, but this approach is currently available only for research purposes.^33^

Retrospective assessment of adherence to medication usually includes large insurance claims-based datasets.^7,8,14,15^ Prospective approaches allow active data collection from participants/medical records during the study, verification of observed information, and collection of specific information of interest not commonly captured in retrospective datasets.^8,19,34^ Prospective assessment of adherence to medication includes objective and subjective methods. Of note, some objective methods (e.g. direct observation; *Table 1*) may facilitate so-called performance bias, such as the Hawthorne effect, when patients adjust their behaviour because of being observed.^19^

Patient-reported assessment enables the examination of subjective constructs (e.g. patients’ opinions, views, experiences, and understanding) using diary records, interviews, or validated survey instruments, which can also be administered to carers or clinicians (*Table 1*).^8,21^ Validity of an instrument refers to its ability to measure the outcome(s) of interest in the population of interest, and the instrument is considered reliable if it produces similar results with multiple administrations.^21^ The most used validated scales for assessing medication adherence are shown in *Table 2*.

**Table 2:** Validated instruments for the assessment of medication adherence

Access	Instrument	Domains of interest	Number of items
No restrictions	Self-reported adherence to medication^23^	Self-reported medication adherence (vs. electronic drug monitoring)	3
	Adherence to refills and medication scale (ARMS)^24^	Health literacy-related factors relevant to adherence to medication in chronic medical conditions	12
	Adherence estimator^25^	(Non)adherence in chronic medical conditions	3
	Medication adherence report scale (MARS-5)^26^	(Non)intentional medication adherence and/or compliance (an improved version of MARS, see below)	5
	Medication adherence self-efficacy scale (MASES)^27^	Self-efficacy and health beliefs	26
Free for non-commercial use	Medication adherence report scale (MARS)^28^	(Non)intentional medication adherence and/or compliance	10
	Brief medication questionnaire (BMQ)^29^	Patient’s perspective on adherence to medication, including potential barriers	5
	Simplified medication adherence questionnaire (SMAQ)^30^	Medication-taking behaviours and adherence	6
Requires licence	Morisky Medication Adherence Scale (four items)^31^	Medication-taking behaviours and potential barriers to adherence	4
	Morisky Medication Adherence Scale (eight items)^32^	Medication-taking behaviours and potential barriers to adherence	8

Accounting for persistence with medication enhances the accuracy of medication adherence assessment. When using a retrospective approach, the rationale(s) for missing prescriptions cannot be captured but incorporating a so-called ‘grace period’ (time since medication was last dispensed after which non-persistence is observed) facilitates the identification of non-persistence in claims data.^8,35^

## Primary non-adherence to oral anticoagulant drugs in atrial fibrillation

Primary non-adherence to medication is a major, yet understudied, healthcare problem. Although widespread implementation of electronic medical records and electronic prescribing has facilitated population-based assessment of primary non-adherence to medication,^10,36–40^ these methodologies cannot differentiate whether a new prescription has not been dispensed or a medication has not commenced the treatment^10^ (*Figure 1*). Some countries are using medical registries that record both prescription and dispensing of medication. The lack of studies assessing the association of primary non-adherence with clinical outcomes and interventions to reduce primary non-adherence represents a major unmet need.

The overall rate of primary non-adherence to medication exceeds 10%.^10,37,38,41,42^ For example, in a systematic review and meta-analysis of *n* = 539 156 patients with various medical conditions [5 randomized controlled trials (RCT), 2 cross-sectional and 26 cohort studies], the overall prevalence of primary non-adherence to medication was 17% [95% confidence interval (CI) 15–20%].^39^ Among patients with AF, primary non-adherence to OAC has been observed in 10–20% of patients.^43–45^

### Determinants of primary non-adherence

Primary non-adherence to medication is multifactorial, involving economic, social, and medical reasons,^46^ and the predictors of primary non-adherence are less well understood. Available data suggest that younger age, multimorbidity, polypharmacy, lack of social support, and low income may be important^38–43,45,47,48^. Additionally, the risk of primary non-adherence has been reported to be higher in asymptomatic medical conditions (compared to symptomatic diseases),^49,50^ discharge to a nursing home,^10,51^ a long distance to the closest pharmacy,^10^ and paper prescriptions (compared with electronic forms),^43,52^ among others.

Among patients with AF, a history of diabetes mellitus, hypertension, or stroke/transient ischaemic attack has been inversely associated with the risk of primary non-adherence to OAC,^44^ and prescription fill rates at 3 months and 1 year were significantly higher in patients prescribed OAC (warfarin) at discharge, compared to those prescribed after discharge (84.5% vs. 12.3% and 91.6% vs. 16.8%, respectively, both *P* < 0.001).^53^

Primary non-adherence to medication often remains undetected in clinical practice. Being unaware of primary non-adherence, the responsible physician may sometimes prescribe treatment intensification where appropriate. More research is needed to better understand the reasons underlying primary non-adherence, improve its detection in routine practice, and develop effective interventions to reduce the prevalence of primary non-adherence in clinical practice.

## Secondary non-adherence to oral anticoagulant drugs in atrial fibrillation

### Prevalence of secondary non-adherence and non-persistence to oral anticoagulant therapy

Secondary non-adherence to OAC represents an important treatment challenge. Whereas an adherence of ≥80% is conventionally considered a good adherence,^54,55^ in a systematic review and meta-analysis of 30 observational studies with a total of *n* = 593 683 participants with AF, the pooled proportion of adherent patients was 63% and 70% at 6 and 12 months after, respectively.^5^

Regarding specific OAC drugs, adherence at 1 year (within the same cohort) with apixaban and rivaroxaban was broadly comparable (74% and 73%), slightly but significantly lower with dabigatran (65%) and lowest with warfarin (50%).^5^ Non-adherence was more prevalent among OAC-naïve compared with OAC-experienced patients.^5,56–58^

Another very comprehensive systematic review and meta-analysis [48 studies (mostly using prescription claims datasets), *n* = 595 784 patients taking a DOAC and *n* = 283 182 patients taking a VKA]^3^ reported findings that are broadly comparable with the previous meta-analysis^5^—a pooled overall proportion of good adherence to OAC of 66% (95% CI 63–70%), to DOACs as a class of 67% (95% CI 61–72%), and to apixaban 71% (95% CI 64–78%), rivaroxaban 70% (95% CI 64–75%) and dabigatran 60% (95% CI 52–68%)^3^. Notably, the latter report^3^ provided additional information that is potentially relevant for future action plans, including persistence with OAC therapy and considerable regional differences in adherence and persistence to OAC therapy.

The overall pooled proportion of persistence to OAC was 69% (95% CI 65–72%), to DOACs as a class was 69% (95% CI 60–77%), and to apixaban 74% (95% CI 69–80%), rivaroxaban 72% (95% CI 68–77%), and dabigatran 62% (95% CI 57–67%; *P* = 0.006).^3^ The overall pooled proportion of good adherence to OAC was significantly lower in North America (63%; 95% CI 60–66%) than Europe (74%; 95% CI 66–82%; *P* = 0.01).^3^ The overall pooled persistence to OAC was the highest in Europe (74%; 95% CI 71–76%), lower in North America (65%; 95% CI 59–70%), and the lowest in Asia/Oceania (49%; 95% CI 36–62%).^3^

Available evidence mostly refers to a ‘mid-term’ adherence for up to 12 months after OAC prescription, whereas the adherence to OAC during over longer periods (i.e. persistence to OAC) is less well known. A recently published study retrospectively assessed long-term patterns of adherence to DOAC therapy over a 3.5-year period in an administrative dataset of 18 920 patients with newly diagnosed AF using the monthly proportion of days covered and a group-based trajectory modelling approach.^59^ Three distinct adherence trajectories were identified—consistent adherence (85.2% of patients), early discontinuation (within 6 months of index date, 10.6%), and gradually declining adherence (4.2%), which could help in selecting groups for tailored adherence interventions.^59^

### Determinants of secondary non-adherence

**Figure 2:**
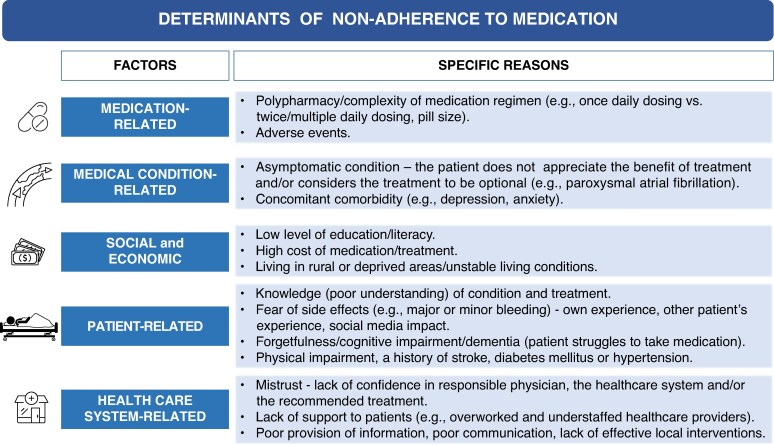
Factors associated with non-adherence to medication.^5,61,62^

Multiple factors have been reported to be significantly associated with (non)adherence^5,60^ (*Figure 2*). Of note, conflicting findings were reported for female sex,^61–63^ age,^56,57,61–64^ baseline risk of stroke,^61,63^ multimorbidity,^57,58^ and polypharmacy.^5,57^ Factors significantly associated with early discontinuation of DOAC included a CHA_2_DS_2_-VASc score of 0–1 (of note, sometimes early OAC discontinuation is justified in low-risk patients such as, for example, patients with a CHA_2_DS_2_-VASc score of 0 receiving OAC peri-procedurally, before and after elective cardioversion or catheter ablation for AF), impaired renal function, antiarrhythmic drug use at baseline, DOAC first prescribed by a non-cardiologist, dementia, and history of falls with injury, while the risk of bleeding (as estimated using the HAS-BLED score) was not significantly associated with the long-term adherence trajectories.^59^

Treatment with OAC is commonly withheld in AF patients at high risk of or with a history of falls, owing to a fear of intracerebral bleeding following a potential fall. A recent study in the UK, for example, reported that older patients with AF and a history of falls were 17% less likely to receive OAC than those with no prior falls.^65^ Importantly, such treatment decisions are often subjective and not based on evidence. Indeed, a large observational study using US Medicare data, for example, reported that the use of DOAC was associated with a 43% risk reduction regarding intracerebral bleeding compared with warfarin in patients at high risk of falls,^66^ while a Markov decision analysis showed that the risk of intracerebral haemorrhage would outweigh the benefits of warfarin therapy in elderly patients (>65 years) with AF only if they fell about 295 times or more per year.^67^ International guidelines for AF management explicitly advise that ‘a history of falls is not an independent predictor of bleeding on an OAC’ and an increased risk of falls does not outweigh the benefits of anticoagulation in older patients.^68^

Notably, patients with AF and general practitioners sometimes overestimate the significance of minor bleeding events (e.g. nose bleeding, excessive bruising), which may result in OAC discontinuation.^69^

In a recently published cohort study of non-anticoagulated patients with AF, the five leading (not mutually exclusive) reasons for not prescribing OAC cited by responsible physicians were low AF burden/successful rhythm control (34.0%), patient refusal (33.3%), estimated low risk of stroke (25.2%), the risk of fall (21.4%), and high bleeding risk (20.4%).^70^

Among participating patients, over 50% were worried about stroke risk (12.3% extremely worried, 33.8% somewhat worried) and/or bleeding events (29.6% extremely worried, 26.6% somewhat worried).^70^ Among patients with a history of prior OAC intake, the most common reasons for stopping OAC were a bleeding event (17.8%), personal preference (9.1%), no further AF (9.1%), or some other reason (13.7%), possibly also including non-affordability of NOACs in some cases. Notably, 65% of non-anticoagulated patients were willing to reconsider OAC use, while only 27% of their physicians would reconsider their initial decision not to prescribe OAC.^70^

These contemporary findings highlight a mismatch of physicians’ and patients’ perceptions and often a patient’s exaggerated impression of OAC-related risk of bleeding, thus emphasizing the need for educational interventions for both physicians and patients and shared treatment decision-making.

### Consequences of non-adherence to oral anticoagulant therapy

The clinical impact of (non)adherence to OAC is summarized in *Table 3*. The economic impacts (i.e. significantly more frequent inpatient and emergency room encounters, longer length of stay, and significantly higher annual adjusted per-patient medical cost in non-adherent compared with adherent patients) have also been reported.^74,75^

**Table 3:** The clinical impact of (non)adherence to OAC in patients with AF

Clinical outcome	Time point	aHR	95% CI	OAC class	Study
Ischaemic stroke	At 12 months	1.50	1.30–1.73	DOACs	Alberts *et al*.^71^
	At 6 months At 12 months	1.82 2.08	1.24–2.67 1.11–3.89	DOACs	Deshpande *et al*.^72^
	At 12 months	1.13	0.97–1.33	Dabigatran	Shore *et al*.^73^
Composite of stroke or death	At 6 months	1.07	1.03–1.12^a^	Dabigatran	Borne *et al*.^56^
	At 12 months	1.13	1.07–1.19^b^	Dabigatran	Shore et al.^73^
Myocardial infarction	At 12 months	0.97	0.78–1.21^c^	Dabigatran	Shore *et al*.^73^
DVT/PE	At 6 months At 12 months	2.12 5.39	1.19–3.78 1.78–16.30	DOACs	Deshpande *et al*.^72^
Non-fatal bleeding	At 12 months	1.04	0.94–1.14^c^	Dabigatran	Shore *et al*.^73^
Major bleeding	At 6 months At 12 months	1.06 0.88	0.82–1.37 0.57–1.36	DOACS	Deshpande *et al*.^72^

aHR, adjusted hazard ratio; CI, confidence interval; OAC, oral anticoagulant; DOAC, direct oral anticoagulant; DVT, deep venous thrombosis; PE, pulmonary embolism.

^**a**^Per 0.1 decrease in the proportion of days covered (PDC).

^b^Per 10% decrease in PDC.

^c^Per 10% increase in PDC.

Noteworthy, a study from Canada addressed the impact of persistence to OAC on clinical outcomes and reported a significant association of non-persistence to dabigatran and rivaroxaban at 6 and 12 months with increased risk of stroke and a composite of all-cause mortality and stroke/transient ischaemic attack.^76^

An analysis of 431 patients admitted to a tertiary hospital with acute ischaemic stroke during 6 months revealed that in 2.6% of patients, i.e. one in every 38 stroke cases had OAC discontinuation within 120 days prior to admission.^77^ In this case series, patients with stroke after OAC discontinuation were older, had more comorbidities, and experienced more severe strokes, with higher mortality and morbidity.

Early discontinuation of DOAC (within 6 months of index prescription) was significantly associated with a two-fold greater risk of thromboembolic events compared with consistent adherence (rate ratio 2.2; 95% CI 1.4–3.5) during follow-up of >12 months, but not within the first 12 months (rate ratio 0.9; 95% CI 0.5–1.4).^59^ Thromboembolic risk with a gradually decreasing adherence pattern was comparable to the risk with consistent adherence to DOAC. Major bleeding event rates, however, were significantly greater with consistent adherence in comparison to early discontinuation patterns, mostly owing to bleeding events observed within the first 12 months of follow-up, thus suggesting the need for careful clinical monitoring even in consistently adherent patients, since the benefit of stroke risk reduction overweighs the harm from bleeding in these patients.^59^

Overall, published observational evidence is mostly obtained retrospectively, using datasets from a few developed countries where proper infrastructure enables systematic data collection, and the most commonly reported measures of adherence to OAC were PDC and MPR at 6 months or 1 year after the index date.^5^ Whether such information can be translated to AF cohorts in developing countries remains unknown.

#### Optimal adherence threshold for oral anticoagulant therapy in atrial fibrillation patients

The medication adherence cut-off of 80% or more has been empirically derived largely from studies on adherence to antihypertensive medications and may not necessarily be valid for OAC therapy, as being minimally evaluated for its clinical relevance in AF patients.^78,79^ Indeed, previous studies of OAC thresholds in AF patients mostly have not properly validated the association of conventional good adherence threshold of ≥80% with major clinical outcomes.

Notably, in a most recent administrative dataset-based study using machine learning (Preprint: Safari A, et al. DOI: 10.1101/2025.06.25329171), optimal OAC adherence thresholds associated with lower risk of clinical outcomes were higher than conventionally assumed—the optimal adherence threshold for VKAs was 85–95% and at least 90% for DOACs. These findings suggest that updating the definition of good adherence to OAC for thromboprophylaxis in AF should be considered.

## Interventions to improve adherence to oral anticoagulant therapy in atrial fibrillation

**Figure 3:**
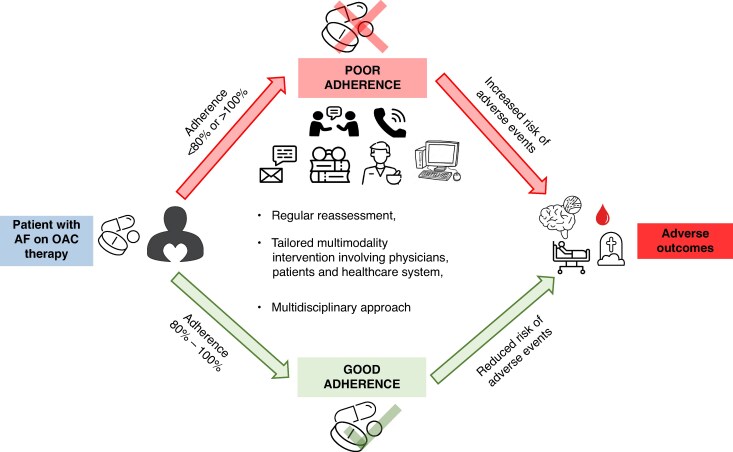
Adherence to medication. AF, atrial fibrillation; OAC, oral anticoagulant.

Regular assessment of adherence to OAC (*Figure 3*) should be an integral part of optimal management of patients with AF, and underlying factors and patterns of non-adherence should be identified in each non-adherent patient to facilitate the implementation of an individualized strategy to improve adherence and patient outcome.

### Patient-centred interventions

#### Shared decision-making with active patient involvement, patient education/counselling, and behavioural interventions

A good physician–patient communication is highly relevant for optimal adherence to OAC therapy.^80,81^ Patients need to feel reassured about the diagnosis and treatment and (mostly) want to be actively involved in the decision-making process. Understanding the individual patient’s values, goals, and preferences regarding their own health allows healthcare professionals to address any concerns about OAC and potential misconceptions. A patient’s perception of the risks of stroke, bleeding, and death, as well as the treatment burden (both overall and OAC-related),^82^ needs to be thoroughly assessed and respected.^83^

A patient’s participation in treatment decision and adherence to agreed treatment is influenced by the patient’s knowledge of AF, stroke, OAC-related risk of bleeding, and drug-specific information, and multiple knowledge gaps have been observed.^84^ Patients need a detailed explanation about AF and their overall medical condition, treatment options (including the risks and benefits of specific therapy, availability of antidote, etc.), how to adhere to OAC (and what to do in case of OAC intake error), and potential consequences of poor adherence to OAC therapy. The information should be provided using appropriate language and various formats, and confirmation that the patient understood the information should be obtained.^6^

The fear of bleeding is still highly relevant for patient adherence to OAC.^85^ Even the possibility of minor bleeding (such as nose bleeding, gum bleeding when brushing teeth, increased bruising, etc.) concerned a considerable proportion (29.3%) of patients taking OAC.^86^ Minor bleeds have been reported to be significantly associated with increased risk of inappropriate OAC discontinuation [hazard ratio (HR) 1.9; 95% CI 1.6–2.2].^69^ This and many other barriers to patient adherence to OAC can be resolved with patient education and shared informed decision-making.

Despite the intuitive potential benefit of patient education and behavioural interventions regarding adherence to OAC, robust evidence is lacking, and available data are conflicting.^87–94^

#### Medication intake monitoring tools and mobile health applications

Various aids/devices for medication intake monitoring (*Table 1*), with or without electronic verification of drug intake and feedback, have been explored and have shown a noteworthy potential to improve adherence to OAC.^6,91,95^ Nevertheless, the long-term effectiveness of these tools is less well known, and a specific tool may not be optimal for all patients or healthcare settings.^95^

Mobile Health technology (mHealth) has great potential to improve adherence to medication, including OAC,^96,97^ notwithstanding the considerable variability among mobile apps used in the management of patients with AF regarding the design, content, and the delivery of intervention(s).^98^ The mAFA app used in the mAFA-II prospective cluster randomized trial resulted in improved clinical outcomes in the intervention arm (based on the ABC pathway) compared to usual care, with >70% adherence and >90% persistence at 1 year.^99,100^ In the ADHERE-App randomized trial (evaluating the effectiveness of a smartphone app in improving adherence to edoxaban in patients with AF), medication adherence measured by pill count at 3 and 6 months did not differ significantly between the two groups. However, the proportion of patients with adequate adherence (≥95%) was significantly higher in the intervention group (76.8% vs. 64.7% at 3 months and 73.9% vs. 61% at 6 months). Patients over 65 years old had the greatest benefit from the intervention, with 81.2% having adequate medication adherence at 6 months vs. 58.9% in the control group (*P* = 0.001).^101^

Mobile apps are generally user-friendly, but more research is needed to inform formal requirements/standardization regarding characteristics and essential active components of AF-management supporting apps.

### Physician- and healthcare system-centred interventions

#### Anticoagulation monitoring services and follow-up of patients

Centralized anticoagulation services (e.g. specialized anticoagulation clinic, nurse- or pharmacist-led AF centres) with a pre-specified patient follow-up plan and involvement of the responsible general practitioner, patient family members, or carers may improve adherence to OAC via regular follow-up visits with adherence reassessment and patient education reinforcement.^60,102–104^ Therefore, optimizing adherence to OAC often depends on the engagement of a multidisciplinary team.^6^

In a recent randomized trial evaluating the effectiveness of a nurse-led behavioural activation intervention lasting 13 weeks in improving health outcomes and decision-making in patients with AF, the intervention group showed significantly greater improvements in health-related quality of life, AF knowledge, and medication adherence immediately post intervention and at 6 months compared with the control group. Furthermore, the proportion of patients with OAC prescription was higher in the intervention group at 6 months [OR 5.87 (1.95–12.33); *P* = 0.012].^105^

#### Physician education and digital clinical decision support systems

Clinicians involved in the management of patients with AF should be constantly updated regarding contemporary management of AF-related stroke risk, and the complexity of those updates should be adjusted to the physician role-appropriate level using various education tools.^6^ In a recent study, for example, an email-based educational intervention targeting primary care physicians with low OAC prescription rates for high-risk patients with AF was associated with a significant decrease in the proportion of untreated patients.^106^

Clinical decision support systems (CDSS) have gradually evolved from educational content distribution and email campaigns towards electronic medical record-embedded notifications to clinicians for upcoming appointments with relevant patients. The effects of multimodality interventions including, for example, electronic medical record notifications combined with email notifications reporting the individual physician’s prescription performance, focus group discussions on knowledge gaps and OAC prescription barriers, as well as in-person or web-based academic detailing of topics related to OAC use in AF, have been compared with usual practice in RCTs^107^ yielding conflicting results. Thus, a systematic review and meta-analysis of nine RCTs addressing the effects of CDSS on adherence to OAC management in patients with AF yielded no significant difference with the use of CDSS compared with routine care regarding the OAC prescription rates, mortality, and bleeding, while the rates of stroke or systemic embolism and myocardial infarction were reduced in the CDSS group.^108^ Of note, barriers to CDSS tools include alert fatigue, time constraints, clinician burnout, workflow interruption, too many recommendations, and irrelevant recommendations, while context-aware CDSS tools could potentially resolve some of these issues.^109–111^

An important limitation of CDSS tools is the lack of patients’ active engagement. Indeed, an RCT comparing a shared decision-making (SDM) tool vs. usual care showed that adding a within-encounter SDM to usual care increased patient involvement and clinician satisfaction without increasing the encounter length.^112^ Nevertheless, the rates of primary and secondary adherence to OAC were comparable in both trial arms (primary and secondary adherence rates were 78%, and 74.1% in the SDM and 81% and 71.6% in the usual care group).^113^

Interventions at the system level should enable and support both patients and clinicians while they actively collaborate in creating plans of care. However, additional large-scale studies are needed to determine the most effective strategies.

At the system level, strategies to improve the affordability of DOACs to patients with AF who need thromboprophylactic therapy must be implemented.^6^

## Anticoagulation formulation

The frequency and complexity of dosing have been shown to affect medication adherence, with once daily dosing being associated with better adherence as compared to twice daily dosing.^79,114–116^ In a large retrospective, insurance claims-based cohort of patients with AF, for example, patients taking rivaroxaban once daily had significantly higher adherence to medication compared to those taking apixaban or dabigatran twice daily, or warfarin (adherence was measured as PDC, and the result was similar whether good adherence was defined as a PDC of ≥0.80 or ≥0.90).^117^

Optimal adherence to OAC is particularly important for effective DOAC therapy, since DOACs have a fast onset and offset of action and shorter half-lives compared to VKAs. While VKA therapy is more tolerant to missing a dose compared with DOACs, studies consistently show lower adherence to VKAs in comparison to DOACs,^3,116^ likely owing to dosing complexity including the need for regular laboratory monitoring of anticoagulation intensity and occasional dose adjustments.

The patterns of non-adherence to OAC therapy in clinical practice are diverse, and missed or delayed dose is the most commonly observed pattern.^118^ Various approaches have been proposed to manage missed or delayed dose in AF patients taking a DOAC, including a recent, individualized model-informed remedial approach based on population pharmacokinetic and pharmacodynamic modelling and simulation with an online dashboard facilitating its use in practice.^119^

A new generation of anticoagulant drugs, inhibitors of Factor XI/XIa, is currently extensively investigated for thromboprophylaxis in several indications including AF.^120^ Factor XI has important role in pathological thrombus formation while being less essential to haemostasis.^121^ Hence, the inhibition of Factor XI/XIa is expected to effectively prevent pathological thrombosis causing less bleeding in comparison to DOAC. However, a recent, prematurely terminated Phase 3 RCT comparing Factor XIa inhibitor asundexian 50 mg once daily with apixaban 5 mg twice daily for stroke prevention in patients with AF (the OCEANIC-AF study)^122^ reported fewer major bleeding events and a higher incidence of stroke or systemic embolism with asundexian during the trial. Therefore, more research is needed to determine whether the Factor XI/XIa inhibition concept may become an option for thromboprophylaxis in patients with AF, what degree of factor XIa inhibition is necessary for effective and safe thromboprophylaxis in AF. Several investigational drug classes inhibit Factor XI/XIa including parenterally (subcutaneously/intravenously) administered monoclonal antibodies and antisense oligonucleotides and are currently undergoing Phase 3 clinical investigation.^123^ Given their half-lives ranging from 14 to 44 days^123^ and parenteral administration, these drugs could improve adherence to anticoagulant therapy in AF patients if approved for stroke prevention in patients with AF.

## Alternatives to oral thromboprophylaxis in patients with atrial fibrillation

Despite the convenience and favourable clinical outcomes associated with DOACs and advances in the science of care for patients with AF, the utilization of OAC in high-risk patients has only modestly increased, and adherence to OAC therapy is still a major clinical concern.

Available evidence suggests that patients would not take their OAC one out of every four days,^3^ and approximately one in three to four patients is poorly adherent to OAC.^3,5^ In addition, around 15% of high-risk OAC-eligible patients with AF would refuse to take OAC for a variety of patient-specific reasons.^108^

**Figure 4:**
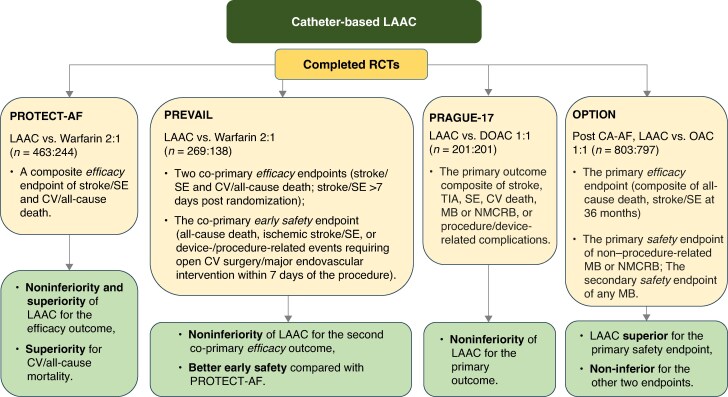
Completed RCTs comparing LAAC with OAC in patients with AF. LAAC, left atrial appendage closure; RCT, randomized clinical trial; SE, systemic embolism; CV, cardiovascular; DOAC, direct oral anticoagulant; TIA, transient ischemic attack, MB, major bleeding; NMCRB, non-major clinically relevant bleeding.

**Figure 5:**
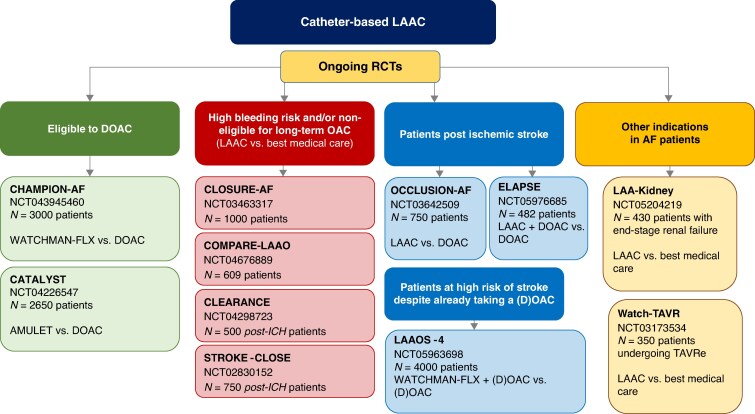
Ongoing RCTs comparing LAAC with OAC/best medical care in patients with AF. LAAC, left atrial appendage closure; RCT, randomized clinical trial; DOAC, direct oral anticoagulant; OAC, oral anticoagulant; ICH, intracranial haemorrhage; AF, atrial fibrillation.

Catheter-based left atrial appendage closure (LAAC) is a viable alternative for effective and safe thromboprophylaxis in AF patients who are non-adherent to OAC and/or have an unacceptably high risk of bleeding.^124^ Completed moderate-sized RCTs support non-inferiority in efficacy of LAAC compared with OAC, especially with warfarin in high-risk AF patients, and recent RCT-based data comparing LAAC with DOACs are encouraging (*Figure 4*).^124,125^ Several ongoing larger-scale RCTs comparing LAAC with DOACs in AF patients with various risk profiles will provide robust evidence further informing the use of LAAC for thromboprophylaxis in patients with AF (*Figure 5*).^124,126^

In a recent systematic review and network meta-analysis of 7 RCTs (*n* = 73 199 patients), the risk of stroke or systemic embolism was comparable between LAAC and DOACs [odds ratio (OR) 1.11; 95% CI 0.71–1.73, *P* = 0.751] and LAAC vs. VKAs (OR 0.93; 95% CI 0.59–1.46, *P* = 0.886). The risk of non-procedural bleeding was significantly lower with LAAC as compared to DOACs (OR 0.55; 95% CI 0.35–0.88, *P* = 0.013) or VKAs (OR 0.44: 95% CI 0.28–0.69, *P* < 0.001). In addition, the risk of all-cause death was significantly lower with LAAC vs. VKAs (OR 0.70; 95% CI 0.53–0.91, *P* = 0.008) and comparable between LAAC and DOACs (OR 0.78; 95% CI 0.59–1.02, *P* = 0.068).^127^

Using LAAC eliminates the need for long-term (lifelong) use of OAC for stroke prevention in most AF patients. Nevertheless, a post-implantation course of antithrombotic therapy is required to cover the period over which the device surface is endothelialized and prevent device-related thrombosis.^124,126^ Based on animal research, endothelialization presumably takes at least 90 days or longer^128^ and, currently, there is no standardized test to determine the degree of LAAC device endothelialization.^126^ Presently, formal recommendations regarding the type and duration of antithrombotic therapy post implantation rely on regulatory device labelling, but post-implantation antithrombotic regimen in a given patient is often modified in clinical practice according to the patient’s stroke and bleeding risk profile, comorbidities, and physician’s preference.^126^

In a recent registry-based observational study of *n* = 31 994 patients undergoing successful LAAC, for example, adherence to the respective RCT-studied full post-procedural treatment protocol was only 12.2%, and alterations in discharge antithrombotic therapy were the most common protocol deviation.^129^ Nevertheless, almost all patients (98.2%) were discharged on an OAC and/or antiplatelet drug. Of note, the risk of any major adverse event (mostly bleeding) at 45 days and 6 months was the highest with VKA + aspirin and significantly lower with DOAC alone or VKA alone, and there was a trend towards a higher rates of device-related thrombus with dual antithrombotic therapy (DAPT) at 45 days.^129^ Ongoing RCTs (*Figure 5*) as well as the FADE-DRT (NCT04502017) and SIMPLAAFY (NCT06521463) trials will help optimizing post-LAAC antithrombotic treatment.^126^

If LAAC is indicated for major bleeding, it must be remembered that short-term antithrombotic therapy will be needed post-implant. In these cases, even a single antiplatelet has been used, with favourable results. Similarly, if the main indication for LAAC is non-adherence, it again should be remembered that adherence to short-term antithrombotic post-implant therapy will be necessary.

## Conclusions

Despite the proven efficacy of DOACs and VKAs in preventing stroke and systemic embolism in high-risk patients with AF, the use of and adherence to OAC in routine clinical practice remain suboptimal. Interventions to improve adherence to OAC are being increasingly developed and tested, but more evidence is needed to inform effective strategies for achieving and maintaining good adherence. However, given the diversity of factors impacting medication adherence, it is unlikely that a single strategy would fit all patients with AF. In patients who remain non-adherent despite intervention, an alternative management of stroke risk using LAAC should be considered, especially in those who refuse anticoagulation because they have suffered from bleeding events or are fearful of such events.

## Data Availability

No new data were generated or analysed in support of this research.
